# A new magnesium sheet alloy with high tensile properties and room-temperature formability

**DOI:** 10.1038/s41598-020-67161-9

**Published:** 2020-06-22

**Authors:** Renhai Shi, Jiashi Miao, Thomas Avey, Alan A. Luo

**Affiliations:** 1 Department of Materials Science & Engineering, The Ohio State University, Columbus, OH USA; 2 Department of Integrated Systems Engineering, The Ohio State University, Columbus, OH USA

**Keywords:** Materials science, Structural materials, Metals and alloys

## Abstract

Lightweight sheet alloys with superior mechanical performance such as high strength, ductility and formability at room temperature (RT) are desirable for high volume automotive applications. However, ductility or formability of metallic alloys at RT are generally inversely related to strength, thereby making it difficult to optimize all three simultaneously. Here we design a new magnesium sheet alloy-ZAXME11100 (Mg-1.0Zn-1.0Al-0.5Ca-0.4Mn-0.2Ce, wt. pct.) via CALPHAD (CALculation of PHAse Diagram) modeling and experimental validation. This new sheet alloy offers an excellent RT formability with a high Index Erichsen (I.E.) value of 7.8 mm in a solution-treated condition (T4), due to its weak and split basal texture and fine grain structure. The new ZAXME 11100 alloy also shows a rapid age-hardening response during post-forming artificial aging treatment at 210 °C for 1 hour (T6), resulting in a significant increase of yield strength from 159 MPa (T4) to 270 MPa (T6). The excellent combination of T4 ductility (31%), T4 formability (7.8 mm) and T6 yield strength (270 MPa) in this new magnesium alloy is comparable to that of common 6xxx series aluminum sheet alloys. Thus, this new magnesium sheet alloy is highly attractive for sheet applications in automotive and other industries.

## Introduction

Steels and 6xxx series aluminum (Al-Mg-Si) alloys are mostly used for automotive body panels due to their excellent formability and subsequent bake hardenability^1,2^. In order to meet the ever-increasing demand for vehicle weight reduction, the use of lighter structural materials has become inevitable in the automotive industry. Magnesium (Mg), the lightest structural metal, has thus attracted considerable attention for applications in automotive and other transportation industries^3,4^. However, compared to 6xxx series aluminum alloys^5,6^ or steels^7^, inferior mechanical properties at RT and high processing cost limit wider applications of commercial Mg sheet alloys such as AZ31 (Mg-3Al-1Zn-0.3Mn) and ZE10 (Mg-1Zn-0.2Ce)^8,9^. This is the well-known issue of the “strength-formability trade-off dilemma” in Mg and other sheet alloys. Thus, it is of great importance to challenge this dilemma and develop a low-cost Mg alloy with both high tensile properties and RT formability.

Magnesium sheet alloys generally have better formability in solution-treated conditions if their annealed texture is weakened; however, they would exhibit low yield strength due to the recovery and recrystallization of their plastically deformed microstructures^10^. Until recently, only a limited number of solution-treated Mg sheet alloys can be substantially strengthened via a bake-hardening treatment (2% pre-strain and aging for 20 min at 170 °C^11^). Such strengthening is attributed to the segregation of solutes to dislocations, as well as the formation of co-clusters of solutes^11,12^ during baking. However, no research has been reported for developing good RT-formable Mg sheet alloys which may be strengthened rapidly and substantially via a low-cost artificial aging treatment rather than via a bake-hardening treatment. The development of heat-treatable Mg sheet alloys with excellent RT-formability and subsequently rapid age-hardenability is therefore a promising approach to overcome the strength and formability trade-off dilemma^13^. The Mg-Al-Zn-Mn-Ca system^11,12,14^ is a promising alloy system to overcome this strength-formability trade-off. Also, trace additions of rare-earth elements like yttrium (Y), neodymium (Nd) and cerium (Ce) can significantly modify the texture and refine grain size to enhance the ductility and formability of wrought Mg alloys. The improvement could be attributed to decreasing the intrinsic stacking fault energy (I_1_ SFE)^15^ to generate <c + a> dislocations, decreasing critical resolved shear stress (CRSS) of pyramidal <c + a > slip^16^, or providing the randomized texture^17^. Thus, the Mg-Zn-Al-Ca-Mn-Ce system is studied with a trace addition of Ce in this work, based on our earlier work^18^.

Extensive research has been reported on discovering mechanical behaviors and improving alloy performance via optimizing alloy composition and thermomechanical processing (TMP)^18–23^. CALPHAD (CALculation of Phase Diagrams) modeling^24–27^, as a part of Integrated Computational Materials Engineering (ICME)^28^ framework, has been successfully applied to study the alloying-processing-structure-property relationships in multicomponent alloy systems. Therefore, CALPHAD simulation is used to design a new Mg-1Zn-1Al-0.5Ca-0.4Mn-0.2Ce alloy (hereafter designed as ZAXME11100) and its optimal TMP to obtain more balanced strength, ductility and formability in this study. TMP (including homogenization, rolling and annealing) is critically important in optimizing the alloying effects for the final mechanical properties of the alloy. For example, conventional homogenization process (at below solidus temperature of the alloy to avoid incipient melting) is inefficient in dissolving second phases from the as-cast microstructure and maximizing the solute concentrations in the Mg matrix, thus achieving the overall alloying effects.

In this paper, CALPHAD simulation is used to design a new homogenization process (with multiple isothermal stages including final stages at temperatures higher than the alloy solidus^18^) for the new alloy, achieving maximum dissolution of alloying elements without incipient melting. The combination of the new alloy design and the multi-stage TMP process leads to the optimum combination of strength, ductility and formability of the new alloy.

## Results and discussions

**Figure 1:**
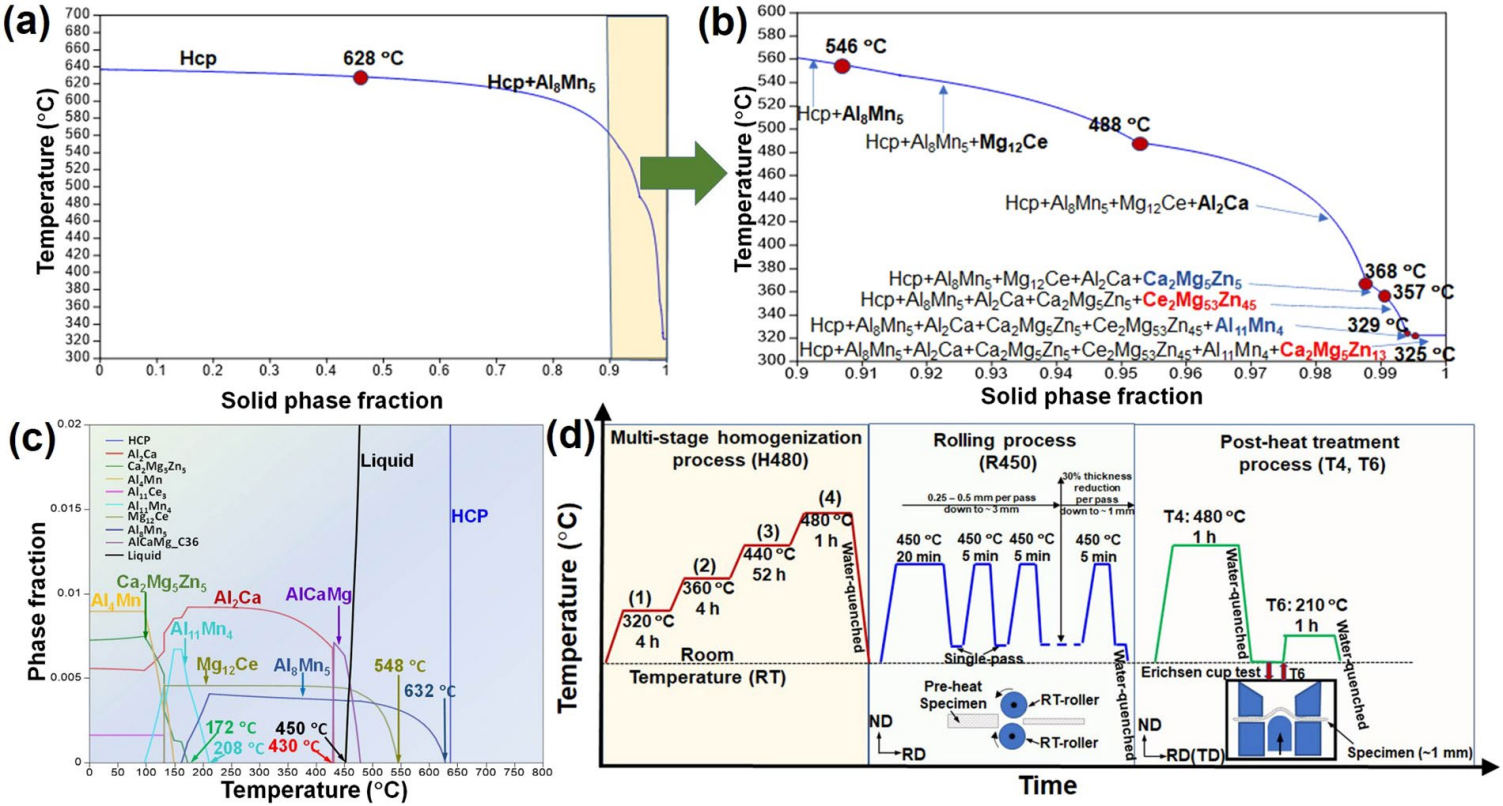
(**a**) Predicted solidification path; (**b**) The enlarged solidification path from the selection in (**a**); (**c**) equilibrium phase fraction vs. temperature calculations; and (**d**) thermomechanical processes for producing ZAXME11100 sheet alloy.

CALPHAD software Thermo-Calc and TCMG5 and MOBMG1 databases^29^ was used to perform the thermodynamic and kinetic modeling of ZAXME11100 alloy. Figure 1(a) shows the solidification path of ZAXME11100 alloy using the classical Scheil model, and an enlarged region near the end of solidification is shown in Fig. 1(b). The results show that HCP (hexagonal closed packed) Mg phase will form as a primary phase at 638 °C, followed by the formation of Al_8_Mn_5_, Mg_12_Ce, Al_2_Ca, Ca_2_Mg_5_Zn_5_, Ce_2_Mg_53_Zn_45_, Al_11_Mn_4_ and Ca_2_Mg_5_Zn_13_ phases at 628 °C, 546 °C, 488 °C, 368 °C, 357 °C, 329 °C and 325 °C, respectively. Figure 1(c) is the equilibrium phase fraction vs. temperature calculation for ZAXME11100 alloy, and the solidus temperature of ZAXME11100 alloy is shown as 450 °C. The calculated formation temperatures of Al_8_Mn_5_ and Mg_12_Ce are 632 °C and 548 °C, respectively, in Fig. 1(c), which are slightly higher than those (628 °C and 546 °C, respectively) of the same phases during cooling (solidification) in Fig. 1(a,b). Similarly, the formation of Al_2_Ca, Ca_2_Mg_5_Zn_5_ and Al_11_Mn_4_ is at 430 °C, 172 °C, and 208 °C, respectively, which is considerably lower than their temperatures (488 °C, 368 °C, and 329 °C) of formation during solidification in Fig. 1(a,b). On the other hand, Ce_2_Mg_53_Zn_45_ and Ca_2_Mg_5_Zn_13_ phases formed during solidification in Fig. 1(b) are not shown in the equilibrium calculation in Fig. 1(c), thus they are assumed to be metastable phases.

Based on these results, a new homogenization profile (designated as H480, thereafter) was designed with four isothermal stages (320 °C for 4 h, 360 °C for 4 h, 440 °C for 52 h and 480 °C for 1 h) to sequentially dissolve the intermetallic phases while avoiding incipient melting. The first stage was designed as 320 °C between the formation temperatures of stable phases (Ca_2_Mg_5_Zn_5_ at 172 °C; and Al_11_Mn_4_ at 208 °C) in Fig. 1(c) and the solidified phases (Ca_2_Mg_5_Zn_5_ at 368 °C; and Al_11_Mn_4_ at 329 °C) in Fig. 1(b), to initially dissolve the stable phases (Ca_2_Mg_5_Zn_5_ and Al_11_Mn_4_) and the metastable phases (Ce_2_Mg_53_Zn_45_ and Ca_2_Mg_5_Zn_13_). The second stage was done at 360 °C for 4 h to further dissolve the phases with higher melting temperatures, such as Ca_2_Mg_5_Zn_5_ phase (368 °C) in Fig. 1(b) and also reduce the micro-segregation of alloying elements.

The third stage was carried out at 440 °C for 52 h between the formation temperature of Al_2_Ca (430 °C) in Fig. 1(c) and (488 °C) in Fig. 1(b), to dissolve Al_2_Ca phase and further homogenize the alloying elements. Figure 1(c) shows a small fraction of liquid at temperatures above the solidus 450 °C. Also, a new phase AlCaMg would possibly form during this stage since it is thermodynamically stable between 430 °C and 478 °C in Fig. 1(c). Thus, the fourth stage was chosen as 480 °C for 1 h to dissolve AlCaMg and potentially Mg_12_Ce and Al_8_Mn_5_ phases with higher thermal stability up to 548 °C and 632 °C, respectively. However, the liquid phase was not experimentally observed during the fourth stage (480 °C, 1 h). The reason could be: (1) the homogenization time was not long enough for the liquid phase to form; or (2) the amount of the liquid phase formed was too small (<~1%, 480 °C in Fig. 1(c)). Thus, the multi-stage homogenization profile (H480) and rolling process (R450) used in producing the ZAXME 11100 alloy sheet are summarized in Fig. 1(d).

**Figure 2:**
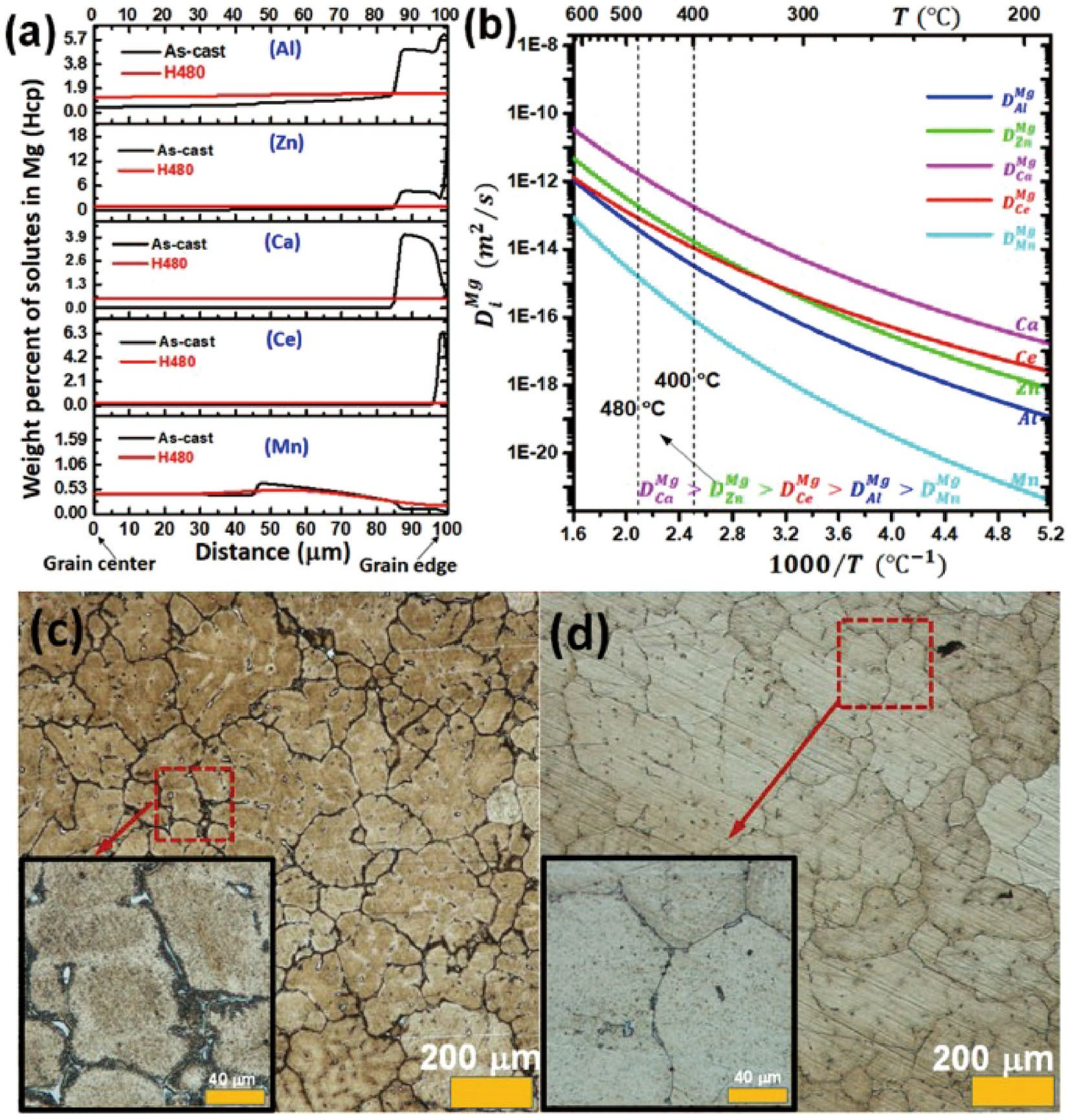
(**a**) Diffusion modeling of solutes Al, Zn, Ca, Ce and Mn in Mg (hcp) using DICTRA software; (**b**) solute diffusion coefficients in Mg (hcp); optical micrographs of (**c**) as-cast condition; and (**d**) after H480 homogenization.

Diffusion-controlled distribution of solutes Al, Zn, Ca, Ce and Mn in Mg matrix was studied using DICTRA^29^ simulation, and Fig. 2(a) plots the calculated solute distributions in both the as-cast (AC) condition and the new H480-homogenized condition. The results demonstrate that the new multi-stage treatment is very effective in dissolving solute elements in Mg grain interiors. Figure 2(b) shows that the diffusion coefficients of Al, Zn, Ca, Mn and Ce solutes in ZAXME11100 alloy are exponentially related to temperature. For example, the diffusion coefficients of the solutes at 480 °C are about 10 times higher than those at 400 °C close to the solidus temperature (450 °C), suggesting that significantly faster homogenization is possible at 480 °C compared to 400 °C (which is below the 450 °C solidus temperature). It is also noted that Al and Mn have the lowest diffusion coefficients among all alloying elements in Mg, which also explains that Al_8_Mn_5_ is the most difficult phase to be dissolved during solution treatment.

Microstructures of ZAXME11100 alloy after as-cast condition and the new homogenization treatment (H480) were observed via optical microscopy and shown in Fig. 2(c,d), respectively. The results showed that the second phase particles near and along the grain boundaries have been essentially dissolved after the new homogenization profile (H480). However, a small amount of residual particles, mostly Al_8_Mn_5_ and a very few Al_2_Ca as identified in TEM, are still visible in Fig. 2(d).

**Figure 3:**
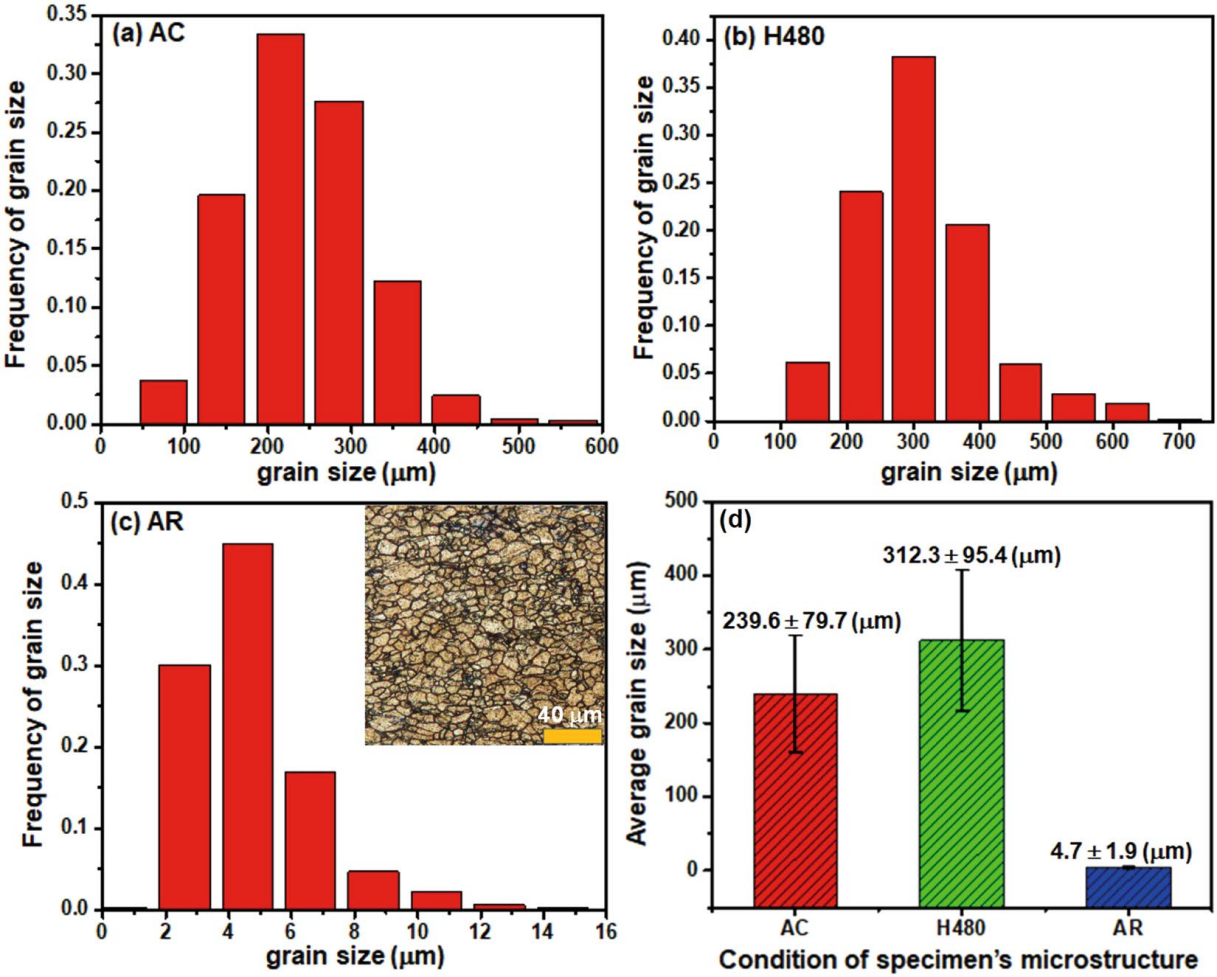
Grain size evolution during different thermomechanical processing: (**a**) AC (as-cast); (**b**) H480 homogenization; (**c**) AR (as-rolled); and (**d**) comparison of average grain sizes at AC, H480 and AR conditions.

The new Mg alloy solutionized at H480 heat treatment has been rolled following the designed rolling process (R450) in Fig. 1(d). The grain size of microstructure from as-cast (AC), H480 and as-rolled (AR) conditions is shown in Fig. 3. It should be noted that (1) the grain size at H480 solution treatment has slightly grown to 312.3 μm, which could be attributed to the inhibition of thermally stable Al_8_Mn_5_ and Al_2_Ca particles formed during the solidification; and (2) the grain size has been significantly decreased to 4.7 μm at as-rolled condition due to recrystallization of the deformed microstructure.

**Figure 4:**
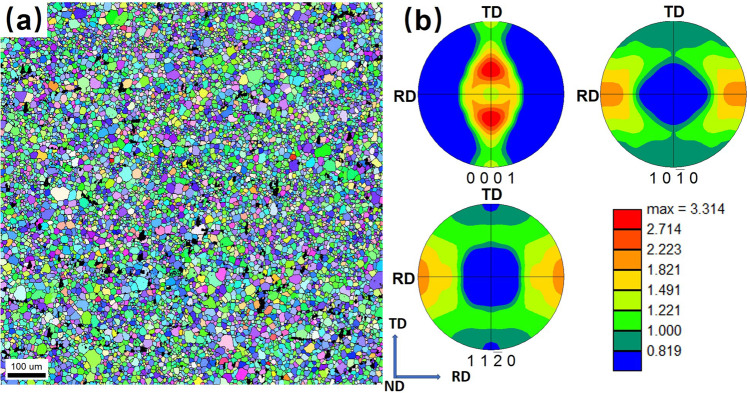
Characterization of the microstructure of ZAXME11100 alloy after solution-treated (T4) at 480 °C for 1 hour: (**a**) EBSD IPF map; (**b**) texture pole figures; (For interpretation of the references to colors in this figure legend, the reader is referred to the web version of this article).

The EBSD inverse pole figure (IPF) map overlaid by large angle grain boundaries with misorientation angles larger than 15 degrees in Fig. 4(a) shows the microstructure of ZAXME11100 alloy after rolling process (R450) followed by the post-forming solution treatment (T4) and water quenching from Fig. 1(d). The average grain size of ZAXME11100 alloy determined via EBSD is about 9.6 μm. It can be seen that the combination of rolling (R450) with the post-forming solution-treatment (T4) yields significant grain refinement in ZAXME11100 alloy. The texture pole figures of solution-treated (T4) microstructure are shown in Fig. 4(b). The microstructure has a weak split basal texture with a maximum intensity of 3.3 mrd. The maximum intensity of basal poles is also tilted by about ±40 degrees away from the normal direction (ND) and toward the transverse direction (TD). Such fine grain size and weakened texture of solution-treated (T4) ZAXME11100 sheet alloy contribute to both high ductility and formability^11,14^.

**Figure 5:**
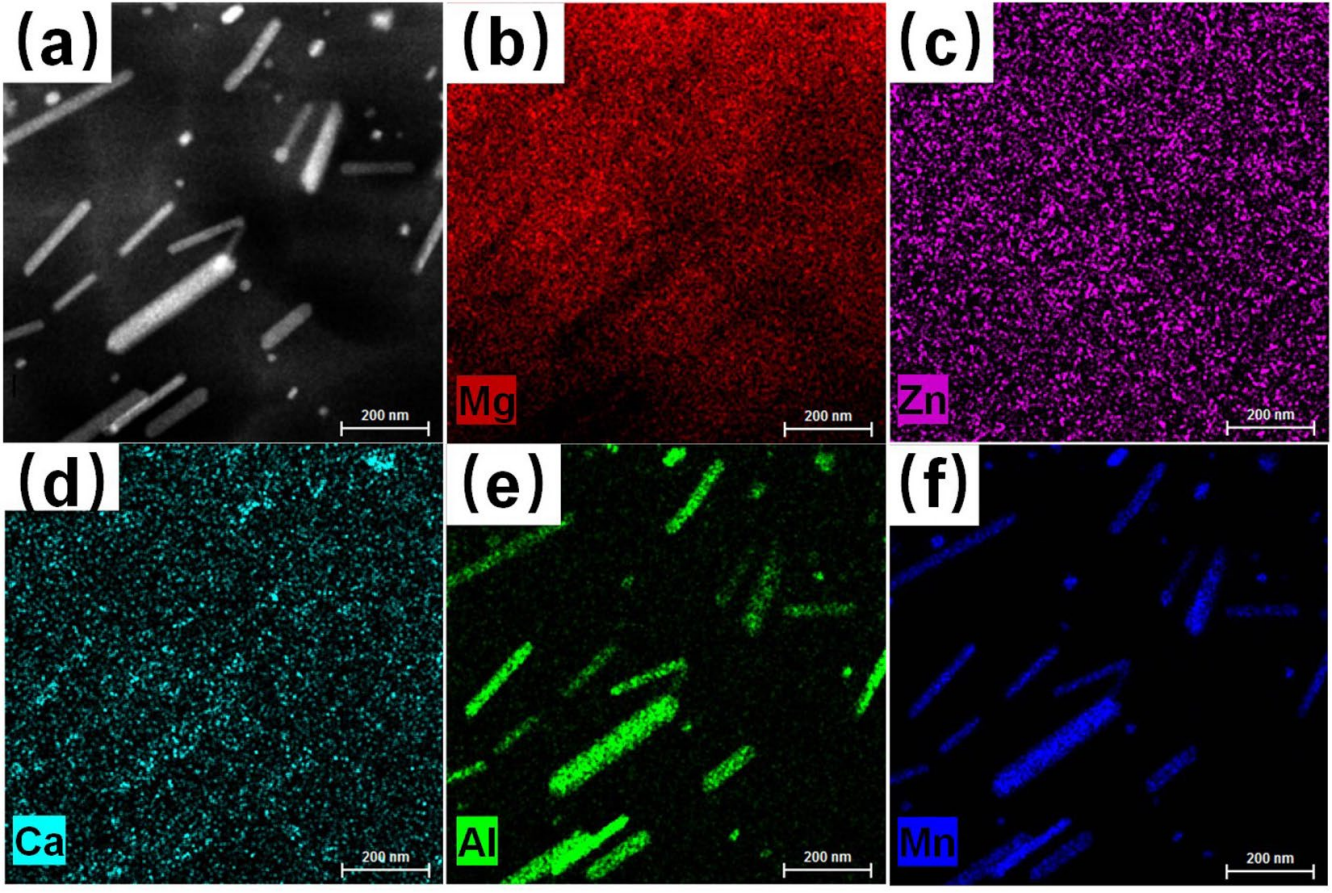
Characterization of the microstructure of ZAXME11100 alloy after solution-treated (T4) at 480 °C for 1 hour: (**a**) HAADF-STEM image; (**b**–**f**) the corresponding STEM-EDS maps for solute elements (Mg-Red, Zn-Magenta, Ca-Turquoise, Al-Green, Mn-Blue).

The high-angle annular dark-field (HAADF) STEM image in Fig. 5(a) shows a high density of intermetallic particles in the post-forming T4 microstructure. STEM energy dispersive spectroscopy (EDS) elemental maps were collected from the region showing in Fig. 5(a). The STEM-EDS elemental maps in Fig. 5(b–f) show that the nano-scale rod precipitates are enriched with Al and Mn elements. Elements Zn, Ca and Ce (not shown in Fig. 5 due to small amount) are homogeneously distributed in the grain interior, suggesting that those alloying elements are dissolved in Mg matrix, hypothesized to form solute co-clusters^11,12^ during subsequent aging heat treatments and thereby contribute to strengthening of the ZAXME11100 alloy.

**Figure 6:**
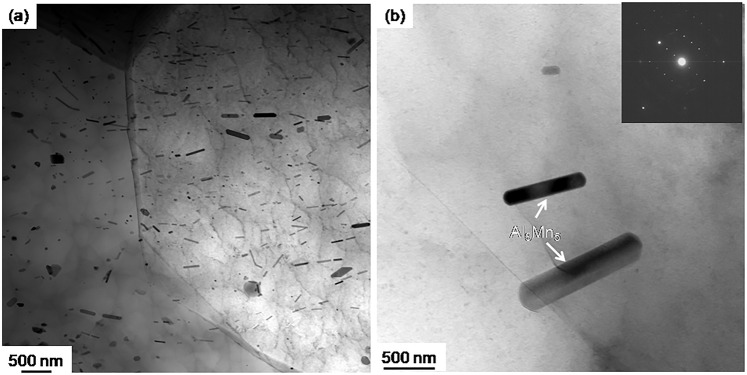
Characterization of the microstructure of ZAXME11100 alloy after solution-treated (T4) at 480 °C for 1 hour: (**a**) BF-STEM image; (**b**) BF-TEM image with an inset selected area diffraction pattern along [113] zone axis of Al_8_Mn_5_ precipitates.

Bright-field (BF) STEM image in Fig. 6(a) reveals that the T4 microstructure consists of a high density of uniformly distributed nano-size rod precipitates randomly distributed both within grain interior and at grain boundaries. Bright field (BF) TEM image with an inset selected diffraction patter in Fig. 6(b) shows that the rod precipitates are Al_8_Mn_5_ phase. As shown in Fig. 1(b,c), Al_8_Mn_5_ starts to form at 628 °C during solidification and melt at 632 °C under the post-heat treatment. It cannot be dissolved during solution treatment at 480 °C. Therefore, Al_8_Mn_5_ can retard grain growth through Zener pinning during annealing at 480 °C, thus contributing to grain refinement. High density and uniformly distributed Al_8_Mn_5_ phase also contribute to the strength of T4 microstructure.

**Figure 7:**
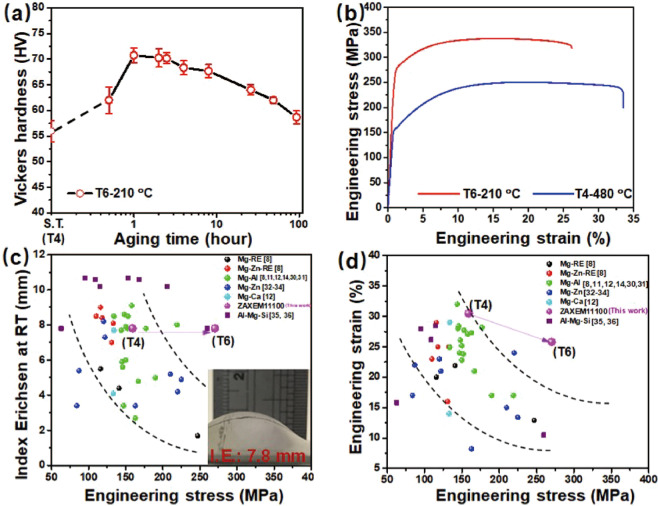
(**a**) Age hardening response at 210 °C; (**b**) tensile curve from the T4 and peak-aged T6 treated ZAXME11100 alloy samples; (**c**) plot of I.E. at RT value as a function of engineering stress; (**d**) plot of engineering strain vs engineering stress at RT.

Figure 7(a) shows the variation in the Vickers hardness of solution-treated (T4) ZAXME11100 sheet alloy under the artificial aging at 210 °C. The ZAXME11100 sheet alloy has a hardness value of 56.0 ± 2.1 HV in the solution treated condition (T4) from Fig. 1(d) and exhibits a rapid age-hardening to a peak hardness of 70.8 ± 1.5 HV for 1 h. The tensile curves obtained from solution-treated (T4) and peak-aged (T6) samples are shown in Fig. 7(b). The mechanical properties of these samples are summarized in Table 1. The T4 treated ZAXME11100 sheet alloy exhibits a moderate yield strength (Y.S.) of 159 MPa and an ultimate tensile strength (U.T.S.) of 253 MPa with an extraordinarily high elongation of 31% and Index Erichsen (I.E.) value of 7.8 mm. After the T6 heat-treatment, the yield strength (Y.S.) and ultimate tensile strength (U.T.S.) have been significantly improved to 270 MPa and 332 MPa with a high elongation of 26%. Figure 7(c,d), respectively, summary the engineering stress as a function of I.E. values as well as engineering strain for various Mg^8,11,12,14,30–34^ and Al^35,36^ sheet alloys. As can been seen, the ZAXME11100 sheet alloy shows good stretch formability in T4 condition, which is close to that reported for 6000 series Al alloy^35,36^. Subsequently the artificial aging (T6) treatment substantially increases the engineering stress with less reduction of ductility, enabling this alloy to have a well-balanced high strength, ductility and formability at room temperature.

**Table 1:** Mechanical properties of ZAXME11100 sheet alloy at room temperature.

Heat treatment	Y.S. (MPa)	U.T.S. (MPa)	Elongation (%)	I.E. (mm)
T4-480 °C, 1 h	159.2 ± 2.5	252.6 ± 2.3	30.5 ± 3.1	7.8 ± 0.1
T6-210 °C, 1 h	270.3 ± 0.4	332.4 ± 5.3	25.8 ± 1.8	

**Figure 8:**
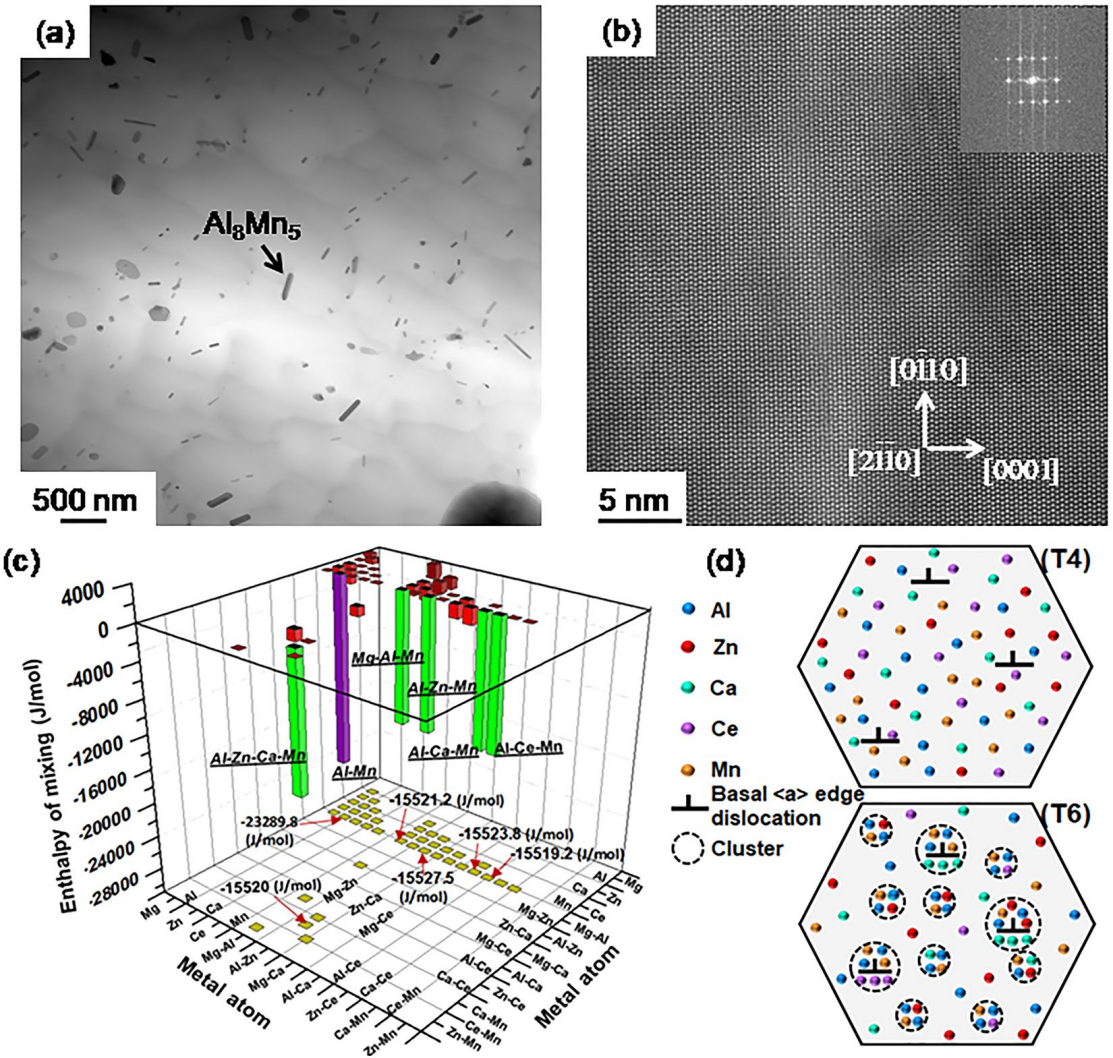
Characterization of microstructure of ZAXME11100 alloy after aging treatment (T6) at 210 °C for 1 hour: (**a**) bright field STEM image; (**b**) atomic resolution HAADF-STEM image; (**c**) projection of mixing enthalpy of various equiatomic pairs in Mg (hcp) matrix; (**d**) top-T4: solute atoms dissolve into Mg (hcp) after solution treatment and bottom-T6: solute atoms not only segregate to basal <a> dislocations but also form small clusters during a short aging time.

The preliminary STEM imaging in Fig. 8(a,b) for the T6 microstructure of ZAXME11100 alloy did not reveal the formation of crystalline precipitate phases, except for the nano-size Al_8_Mn_5_ rods which were seen previously in the solution-treated samples (Fig. 6). This suggests that solutes Al and Mn have the strongest affinity in the ZAXME11100 alloy and that other solutes may not have enough strong affinity to form crystalline phases during aging. Figure 8(c) shows the enthalpy of mixing values of various atomic pairs in the ZAXME11100 system. The calculated result shows that the enthalpies of mixing of Al-Mn (−23289.8 J/mol), Al-Zn-Ca-Mn (−15520 J/mol), Al-Ca-Mn (−15523.8 J/mol), Al-Ce-Mn (−15519.2 J/mol), Al-Zn-Mn (−15527.5 J/mol), and Mg-Al-Mn (−15521.2 J/mol) pairs are significantly negative. Therefore, thermodynamically, there is a strong affinity between the corresponding solutes to form solute clusters or crystalline phases. It should be noted that the Al-Mn pair has the most negative enthalpy of mixing, suggesting that solute pair Al and Mn has a much stronger affinity than other solute pairs, and therefore is most likely to form crystalline precipitates.

Based on results from Fig. 8(a–c), the Al-Mn atomic pair with strongest affinity would form crystalline Al_8_Mn_5_ phase. Other atomic pairs with negative mixing enthalpies may form some clusters^11,12^ without forming distinct observable precipitates during the ageing treatment. Further characterization work is on-going to investigate the strengthening mechanism involved in T6 treatment. Therefore, the significant enhancement of engineering strength after T6 treatment would be attributed to the formation of solute clusters^11,12^ in Fig. 8(d), which should provide a string pinning effect on the basal <a> dislocations. These dislocations need a larger force to break away from the solute atmosphere after aging (T6). Therefore, the combination of designing new alloy and optimizing TMP with CALPHAD methodology has led to a heat-treatable magnesium sheet alloy (ZAXME11100) with excellent RT-formability and high strength, which has overcome the well-known strength-formability dilemma.

## Conclusions

In summary, a new Mg sheet alloy (ZAXME11100: Mg-1Zn-1Al-0.5Ca-0.4Mn-0.2Ce) and a novel thermomechanical process have been developed based on CALPHAD and diffusion modeling in combination with experimental investigation. The new Mg sheet alloy produced via low-cost rolling followed by a multi-stage solution treatment (T4) and conventional aging treatment (T6) has a superior combination of high strength (270 MPa in T6), ductility (31% in T4) and formability (Erichsen Index of 7.8 mm in T4) at room temperature. Microstructure characterization and thermodynamic/kinetic calculations suggest that the following factors contribute to the excellent properties: (1) weak and split basal texture; (2) fine grain structure (average 9.6 μm); and (3) possible formation of solute clusters after a short-time aging treatment (T6) due to the negative mixing enthalpy of various solute pairs. The new ZAXME11100 sheet alloy has successfully overcome the common trade-off relationship between formability/ductility and strength, promising high-volume automotive applications of magnesium sheet components.

## Materials and methods

Magnesium sheet alloy preparation method was reported in a previous publication^18^. Mg-1Zn-1Al-0.5Ca-0.4Mn-0.2Ce (ZAXME11100) alloy was prepared in a steel crucible under a protective gas mixture of CO_2_ and SF_6_, then cast at 750 °C into a steel mold pre-heated to 400 °C to produce 110 × 114 × 18 mm plates. The cast plates were machined into 20 × 28 × 7 mm samples which were homogenized (multi-stage H480) and water-quenched. The homogenized samples were cold-rolled initially to ~3 mm with about 0.25 to 0.5 mm thickness reduction per pass, and then rolled to ~1 mm with 30% thickness reduction per pass. Except for the final rolling pass, the samples were pre-heated at 450 °C for 5 min prior to each pass. Longitudinal tensile samples with a gauge length of 12.5 mm, a width of 5 mm, and a thickness of 1 mm were machined from the as-rolled sheets. Some as-rolled tensile samples were solution-treated at 480 °C for 1 h (T4) followed by water-quenching and then artificial aging at 210 °C for 100 hours in an oil bath. Age hardening responses of the solution-treated samples were measured using a Vickers hardness tester. Tensile tests were conducted at RT on the solution-treated (T4) and peak-aged (T6) samples at a strain rate of 1.8 × 10^−4^ s^−1^. At least three specimens were tested at RT for each temper condition to ensure repeatability. To evaluate the stretch formability of the solution-treated (T4) sheets, Erichsen cupping tests were performed at RT on 60 × 60 mm rectangular samples with a thickness of 1 mm using an Erichsen sheet metal testing machine with a 20 mm diameter hemispherical punch. The punch speed and blank-clamping force were around 6 mm/min and 10 kN, respectively. Graphite lubrication was used on the tool.

The microstructure of the new alloy was characterized using optical microscopy, scanning electron microscopy (SEM), transmission electron microscopy (TEM) and scanning transmission electron microscopy (STEM). The SEM study and electron backscatter diffraction (EBSD) mapping were conducted using a FEI Apreo SEM equipped with a EDAX OIM system. Specimens for TEM analysis were mechanically ground to ~40 μm using a Fischione model 100 dimpler. Perforation of thin TEM foils were conducted in a Fischione model 1010 ion mill operating at a voltage of 5 keV. TEM and STEM investigations were carried out on a FEI Tecnai F20 TEM/STEM microscope operating at 200 kV. STEM-EDS elemental maps were collected using a FEI G2 60-300 TEM/STEM microscope equipped with a Super-X/ChemiSTEM) EDS system operating at 300 kV.
